# Isoflavonoid-Antibiotic Thin Films Fabricated by MAPLE with Improved Resistance to Microbial Colonization

**DOI:** 10.3390/molecules26123634

**Published:** 2021-06-14

**Authors:** Valentina Grumezescu, Irina Negut, Rodica Cristescu, Alexandru Mihai Grumezescu, Alina Maria Holban, Florin Iordache, Mariana Carmen Chifiriuc, Roger J. Narayan, Douglas B. Chrisey

**Affiliations:** 1 Lasers Department, National Institute for Lasers, Plasma and Radiation Physics, 077125 Magurele, Romania; valentina.grumezescu@inflpr.ro (V.G.); negut.irina@inflpr.ro (I.N.); 2 Department of Science and Engineering of Oxide Materials and Nanomaterials, Faculty of Applied Chemistry and Materials Science, Politehnica University of Bucharest, 011061 Bucharest, Romania; grumezescu@yahoo.com; 3 Research Institute of the University of Bucharest–ICUB, University of Bucharest, 050657 Bucharest, Romania; alina_m_h@yahoo.com (A.M.H.); carmen.chifiriuc@gmail.com (M.C.C.); 4 Department of Microbiology and Immunology, Faculty of Biology, University of Bucharest, 077206 Bucharest, Romania; 5 Department of Biochemistry, Faculty of Veterinary Medicine, University of Agronomic Science and Veterinary Medicine, 59 Marasti Boulevard, 011464 Bucharest, Romania; floriniordache84@yahoo.com; 6 Academy of Romanian Scientists, Ilfov no. 3, 50044 Bucharest, Romania; 7 Joint Department of Biomedical Engineering, University of North Carolina and North Carolina State University, Raleigh, NC 27606, USA; roger_narayan@unc.edu; 8 Department of Physics and Engineering Physics, Tulane University, New Orleans, LA 70118, USA; dchrisey@tulane.edu

**Keywords:** luteone, wighteone, isoflavonoid, polyvinylpyrrolidone, ceftriaxone, cefuroxime, pharmaceutic, laser fabrication, matrix-assisted pulsed laser evaporation, antimicrobial resistance

## Abstract

*Staphylococcus aureus* (Gram-positive) and *Pseudomonas aeruginosa* (Gram-negative) bacteria represent major infectious threats in the hospital environment due to their wide distribution, opportunistic behavior, and increasing antibiotic resistance. This study reports on the deposition of polyvinylpyrrolidone/antibiotic/isoflavonoid thin films by the matrix-assisted pulsed laser evaporation (MAPLE) method as anti-adhesion barrier coatings, on biomedical surfaces for improved resistance to microbial colonization. The thin films were characterized by Fourier transform infrared spectroscopy, infrared microscopy, and scanning electron microscopy. In vitro biological assay tests were performed to evaluate the influence of the thin films on the development of biofilms formed by Gram-positive and Gram-negative bacterial strains. In vitro biocompatibility tests were assessed on human endothelial cells examined for up to five days of incubation, via qualitative and quantitative methods. The results of this study revealed that the laser-fabricated coatings are biocompatible and resistant to microbial colonization and biofilm formation, making them successful candidates for biomedical devices and contact surfaces that would otherwise be amenable to contact transmission.

## 1. Introduction

Despite significant medical progress related to the development of antibiotics over the last century, healthcare-associated infectious diseases remain a considerable threat throughout the world [1,2,3]. The most regularly encountered and transmitted healthcare-associated infectious diseases are caused by *Staphylococcus aureus* and *Pseudomonas aeruginosa* [4,5,6]. Even though antibiotics have significantly reduced the occurrence of infectious diseases, the overuse of antibiotics can cause bacteria, viruses, fungi, and parasites to mutate and develop resistance [7,8,9]. One main reason for the ineffectiveness of antibiotic treatments is the development of microbial multicellular attached communities, called biofilms [10,11].

The ability to target multiple pathways involved in biofilm formation and dispersal is highly desirable. Antimicrobial mixtures are used to stimulate pharmaceutical action or accomplish synergistic effects in order to treat mixed bacterial infections in which the participating microorganisms are resistant to multiple antimicrobial agents [12], avoid the occurrence of drug tolerance [13], and minimize drug toxicity [14]. The use of a combination of antibiotics is an interesting approach to develop effective antibiofilm therapies. Moreover, the incorporation of several bacterial resistance mechanism inhibitors and conventional antibiotics has been described in the literature. For example, clavulanic acid has been used in combination with amoxicillin; in addition, sulbactam has been joined with ampicillin and tazobactam has been joined with piperacillin [15]. These combinations could be very efficient against specific types of resistant microbial strains; however, the combinations may exhibit narrow antimicrobial activity spectra. The limitations of currently available antimicrobial agents and the development of drug-resistant strains demand more effective tactics [16,17].

Significant research has been focused on extracts from natural medicinal plants. Plants contain metabolites such as terpenoids, monoterpenoids, flavonoids, alkaloids, saponins, tannins, and phenylpropanoids that exhibit antioxidant and antimicrobial properties [18,19].

**Figure 1:**
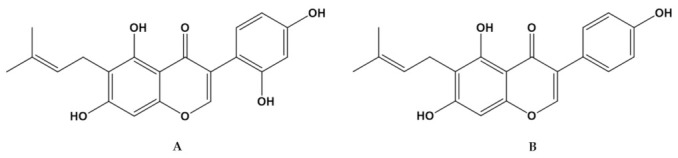
Chemical structures of (**A**) FL (**A**) and (**B)** FW.

Flavones and their subclass known as isoflavones belong to the polyphenolic secondary metabolite family, and these compounds possess extensive pharmaceutical activity. They have been associated with various health benefits, including antioxidant, antiviral, antitumor, antiosteoporotic, estrogenic, and anti-inflammatory. Flavonoids impede the main hallmarks of cancer by inhibiting cell proliferation and growth, triggering apoptosis, or a mixture of these activities [20,21]. Flavonoids and isoflavones have been tested against a panel of pathogenic microorganisms, including Gram-positive and Gram-negative bacteria and fungi. Among these phytochemicals, the natural isoflavonoid known as wighteone, or 5,7,4′-trihydroxy-6-prenylisoflavone, 6-prenylgenistein (FW) (Figure 1, right), showed potent activity against a clinical strain of the Gram-positive organism *S. aureus* [22,23]. In a test against *Escherichia coli*, a Gram-negative organism, wighteone exhibited more significant activity than other classes of isoflavonoids [23,24,25]. Another antifungal isoflavone, luteone, or 5,7,2′,4′-tetrahydroxy-6-prenylisoflavone (FL) (Figure 1, left) [23,26], present in the surface wax of lupin leaves, has been shown to inhibit spore germination and/or germ tube development [27].

When used in conjunction with antibiotics, flavonoids were found to increase antibiotic activity against bacteria. For example, ceftriaxone (CEFTRI) and cefuroxime (CF), two cephalosporin antibiotics with a broad-spectrum activity against Gram-positive and Gram-negative bacteria [28], presented an enhanced activity against both standard strain and clinical isolates of *S. aureus* by association with morin + rutin [29] and sophoraflavanone B [30] flavonoids, respectively. Moreover, several reports have noted that flavonoids and other phytochemical compounds can augment the effect of antibiotics [31,32,33,34,35].

**Figure 2:**
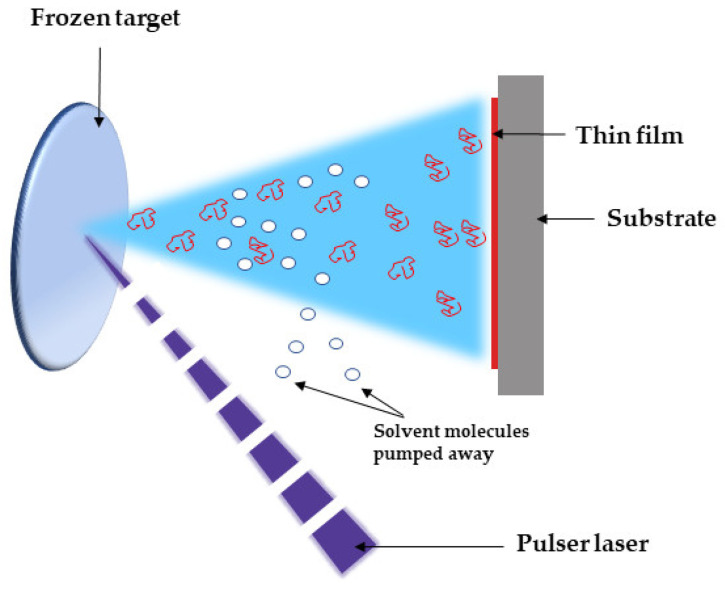
Simplified schematic of the MAPLE process.

Many strategies have been advanced in order to reduce the prevalence of medical device-related infections [36]. One approach to prevent biofilms involves the design of novel medical device surfaces that limit microbial adhesion and/or growth [37]. Biocides that can be integrated into bulk materials (e.g., various polymeric surface coatings) [38,39] have also been considered. These composite materials can be prepared as nanomaterial-based remedies to combat challenging bacterial infections, and they can include the capacity to trick mechanisms related to acquired drug resistance. Moreover, the unique size and physical features of nanomaterials provide them with the capability to target biofilms and defeat resistant infections [40,41]. In this respect, matrix-assisted pulsed laser evaporation (MAPLE) has been successfully applied for the growth of high-quality nanomaterial coatings with useful functionalities for biomedical applications [42,43,44,45,46,47]. MAPLE is an adaptable laser-based processing method that was developed to fabricate new types of bioactive hybrids for use as antimicrobial coatings [48,49]. The MAPLE technique implies a vacuum deposition chamber in which a pulsed laser beam strikes and evaporates a rotating cryogenic target containing a diluted mixture of the complex organic material to be deposited. Prior to the laser ablation, the organic material of interest is dissolved in a light absorbent, high vapor-pressure solvent, and the resulted solution is poured within a target holder and then frozen at the liquid N_2_ temperature (77 K). The matrix (volatile solvent) is pumped away by the vacuum system. The incident laser pulse that sweeps the cryogenic target initiates two photo-thermal processes: (i) the evaporation of the frozen composite target, and (ii) release of the organic material into the chamber, to be collected on the substrates in the form of a thin film (Figure 2) [42,43,44,45,46,47,48,49].

Our previous flavonoid-related work demonstrated that MAPLE is a suitable technique for fabricating biomimetic thin films containing combinations of antimicrobial compounds, including quercetin dihydrate and resveratrol flavonoids, norfloxacin and CF large-spectrum antibiotics [50], amphotericin B and voriconazole [51], systemic antifungal agents, as well as silver nanoparticles [52]. These films showed antibacterial efficiency against Gram-positive and Gram-negative bacterial strains, and this activity was mediated by a contact-killing effect [50,51,52]. An important outcome of this work was to indicate the potential use of quercetin as a substitute for antibiotics to inhibit mature biofilm growth on various substrates. Due to the efficacy of quercetin to target the SARS-CoV-2 3CLpro main protease [53], quercetin flavonoid-based surface coatings can combat COVID-19 contact transmission [54,55].

In the present study, we used matrix-assisted pulsed laser evaporation to deposit combinations of an isoflavonoid (FL or FW), a biopolymer (polyvinylpyrrolidone (PVP)), and an antibiotic (CF or CEFTRI) as thin films. We investigated their chemical structure by Fourier transform infrared (FT-IR) spectroscopy and infrared microscopy (IRM), as well as their surface morphology by scanning electron microscopy (SEM). In addition, their antimicrobial activity against both Gram-positive and Gram-negative bacteria was assessed in view of the possible use of these systems for the development of novel antimicrobial strategies. Moreover, the biocompatibility of the thin films was evaluated.

## 2. Results and Discussions

### 2.1. FT-IR and IRM

**Figure 3:**
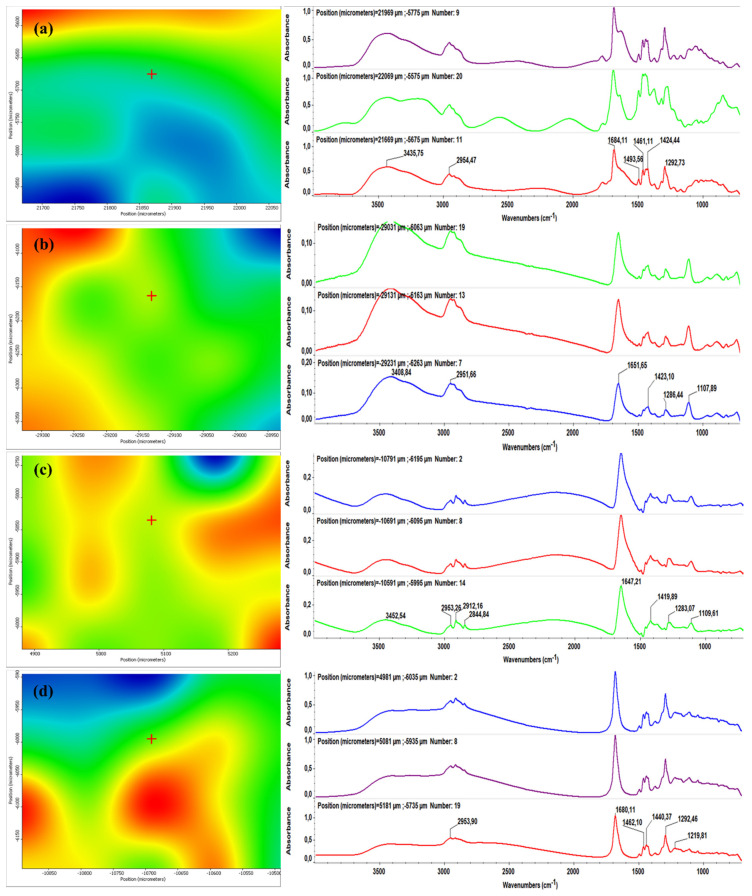
Second derivate IR mappings (left) and FT-IR spectra (right) of CF/PVP/FL (**a**) drop-cast, and thin films deposited at a laser fluence of (**b**) 100 mJ/cm^2^, (**c**) 200 mJ/cm^2^, and (**d**) 300 mJ/cm^2^.

**Figure 4:**
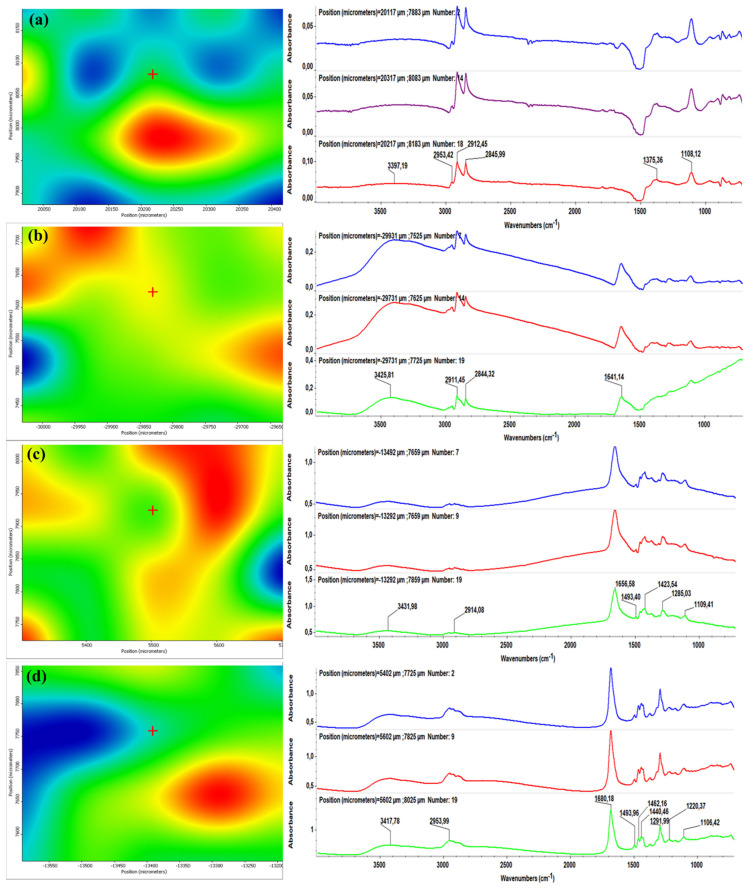
Second derivate IR mappings (left) and FT-IR spectra (right) of CEFTRI/PVP/FW (**a**) drop-cast, and thin films deposited at a laser fluence of (**b**) 100 mJ/cm^2^, (**c**) 200 mJ/cm^2^, and (**d**) 300 mJ/cm^2^.

Figure 3 and Figure 4 illustrate the second derivative infrared (IR) micrographs (left columns) of CF/PVP/FL (Figure 3) and CEFTRI/PVP/FW (Figure 4) coatings respectively, and their corresponding FT-IR spectra (right columns). The above-mentioned figures contain the infrared data of drop-cast samples (Figure 3a and Figure 4a) and MAPLE coatings obtained at laser fluences of 100, 200, and 300 mJ/cm^2^ (Figure 3b–d and Figure 4b–d).

The color modification of the IR maps from Figure 3 and Figure 4 (left images) is correlated to the variation of absorbance from blue to red (these colors correspond to extreme lowest and highest absorbance intensities, respectively). It can be generally noticed that the infrared micrographs of all of the drop-cast samples (Figure 3a and Figure 4a) have an abundance of blue areas that indicate the reduced amount of material onto the substrates. In the case of MAPLE-deposited samples (Figure 3b–d and Figure 4b–d), the blue areas are more reduced than the drop-cast specimens, indicating a higher amount of deposited material. It can be noticed that the laser fluence value of 200 mJ/cm^2^ provides the most efficient transfer of composite materials (Figure 3c and Figure 4c), as evidenced by the abundance of red and orange areas. Additionally, the infrared spectra corresponding to the drop-cast and MAPLE coatings obtained at 200 mJ/cm^2^ (Figure 3a,c, and Figure 4a,c, right columns) are very similar, and this result indicates that the MAPLE deposition process performed at this particular laser fluence did not affect the chemical composition of materials. In comparison to drop-cast samples, samples made at a high laser fluence (300 mJ/cm^2^) showed relevant modifications. In the case of the lowest laser fluence (100 mJ/cm^2^), a significant decrease in the intensity of the main infrared maximum was observed. This result could be related to the insufficient transfer of the composite materials. On the other hand, the highest laser fluence (300 mJ/cm^2^) showed loss and position shifting of some infrared maxima, and these phenomena are related to the degradation of composite materials.

It can be concluded that neither of the laser fluence values of 100 and 300 mJ/cm^2^ represent a suitable choice for processing CF/PVP/FL and CEFTRI/PVP/FW materials. In terms of compositional integrity and optimal material transfer, the middle laser fluence value represented the best choice for MAPLE processing of composite materials. Therefore, we decided to continue this study using 200 mJ/cm^2^ laser fluence for these antibiotic-free materials.

**Figure 5:**
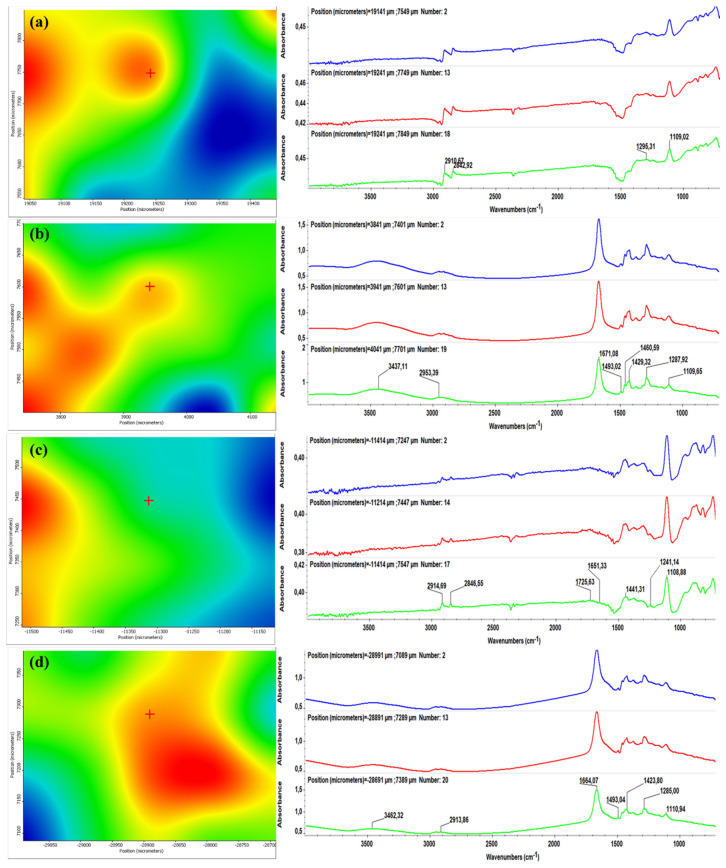
Second derivate IR mappings (left) and FT-IR spectra (right) of (**a**) PVP/FL drop-cast, (**b**) PVP/FL thin films deposited at 200 mJ/cm^2^ laser fluence, (**c**) PVP/FW drop-cast, and (**d**) PVP/FW thin films deposited at 200 mJ/cm^2^ laser fluence.

Figure 5 contains the IR micrographs (left column) and the corresponding IR spectra (right column) of PVP/FL (Figure 5a,b) and PVP/FW (Figure 5c,d) coatings. The images (a) and (c) correspond to the drop-cast samples, while the images (b) and (d) correspond to the MAPLE coatings fabricated at 200 mJ/cm^2^ laser fluence. When compared to the antibiotic-loaded coatings (as previously discussed), the infrared data corresponding to MAPLE-deposited PVP/FL and PVP/FW samples mainly contain the same absorbance maxima. No changes to the spectral features were observed. In comparison to the antibiotic-loaded coatings, the slight intensity variations observed in the infrared bands identified in Figure 5 may indicate that only weak physical interactions occurred between the antibiotics and the PVP/isoflavonoid compounds.

In case of IR maps, at 200 mJ/cm^2^ laser fluence, the drop-cast and MAPLE thin-film FT-IR spectra show a very close resemblance to the corresponding standard (non-thin film) spectra in the characteristic fingerprint region.

For this, we identified the characteristic peaks of thin-film drop-cast and MAPLE samples and compared them to those assigned to PVP, CF, CEFTRI, FL, and FW standard functional chemical groups (fingerprints) in Figure 3, Figure 4 and Figure 5 (right side).

The characteristic PVP FT-IR absorption features are visible in Figure 3, Figure 4 and Figure 5 (right side). Thus, the peak at ~3462 cm^−1^ is indicative of the O-H stretching [56]. The peaks at ~2953 and ~1671 cm^−1^ prove the existence of CH_2_ asymmetric stretching and stretching of C-O, respectively [56]. The C-H bending and CH_2_ wagging were observed at ~1429 and ~1287 cm^−1^, respectively [56].

The presence of CF in the FT-IR spectra (Figure 3, right side) is indicated by the bands in the region of: ~(1680–1648) cm^−1^ assigned to C=O group, ~(1200–1110) cm^−1^ for C–C group, and ~(3500–3400) cm^−1^ designated for NH (amide group) [57].

Figure 4 (right side) reveals typical FT-IR spectra that contain a CEFTRI characteristic peak at ~3431 cm^−1^ that is ascribed to N-H stretching vibration of the H-bonded amide group. The presence of the band centered at ~2953 cm^−1^ is assigned to stretching vibrations of C–H groups. The peaks at ~1740 and ~1649 cm^−1^ are attributed to C=O stretching vibration and at ~1493 cm^−1^ to C-N stretching vibration. The CEFTRI antibiotic shows characteristic absorptions at ~1748 and ~1554 cm^−1^ corresponding to β lactam C=O and C=N, respectively [58]. The peak at ~1106 cm^−1^ could be accredited to the stretching vibrations of C–O.

FT-IR spectra on flavonoids have been revised. The fingerprints (characteristic chemical bonds) that are common for the flavonoid structures include C=O bond (1630–1665) cm^−1^, C-O bond (1000–1300) cm^−1^, and in-plane deformation vibrations of C-H (600–980) cm^−1^ [59]. Other studies have identified the OH group at 3300, 2970, and 2856 cm^−1^, and C=C at 1644 cm^−1^ [60]. In our case, FL has the absorption peak around the region centered at 1642 cm^−1^, assignable to C=O carbonyl function (Figure 3 and Figure 5a,b, right side) [61]. Additionally, FW has two absorption peaks at 3400 and 1650 cm^−1^ that are visible in Figure 4 and Figure 5c,d (right side), being in good agreement with the literature [62].

### 2.2. SEM

**Figure 6:**
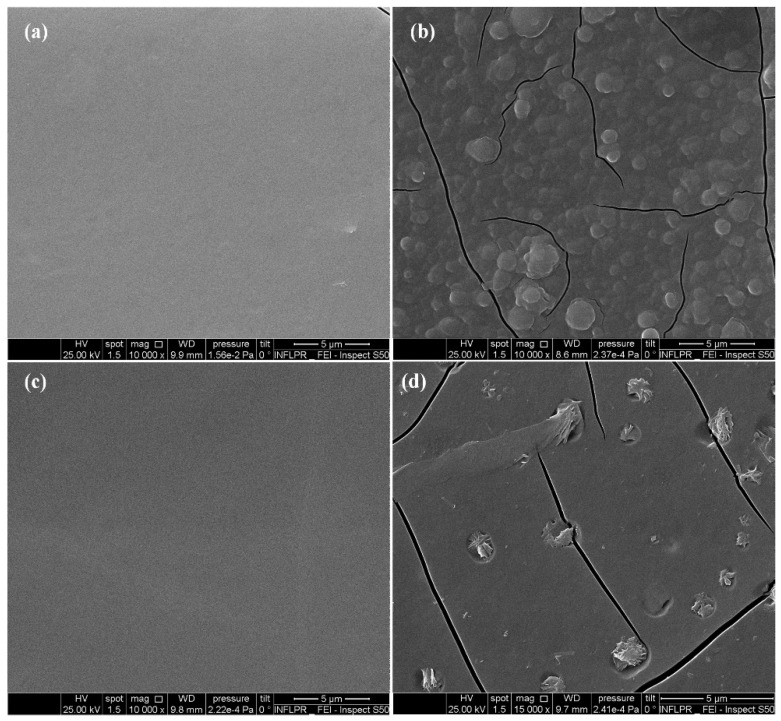
SEM images of thin films deposited at 200 mJ/cm^2^ for (**a**) PVP/FL, (**b**) CF/PVP/FL, (**c**) PVP/FW, and (**d**) CEFTRI/PVP/FW.

The surface morphology of thin films (with mechanical cracks) was evaluated using SEM (Figure 6). It can be observed that all SEM images show uniform surfaces. When comparing Figure 6a,b, some morphological changes can be observed. According to Figure 6b, individual globular structures embedded into the polymeric thin film can be identified. The presence of these structures can be attributed to the addition of CF to the mixes. As it can be noticed (by comparing Figure 6c,d), the morphology of thin films is modified by the addition of CEFTRI. In particular, on the surface of thin films of CEFTRI/PVP/FW, one can observe the presence of scattered flake-like structures.

### 2.3. Antimicrobial and Antibiofilm Evaluation

**Figure 7:**
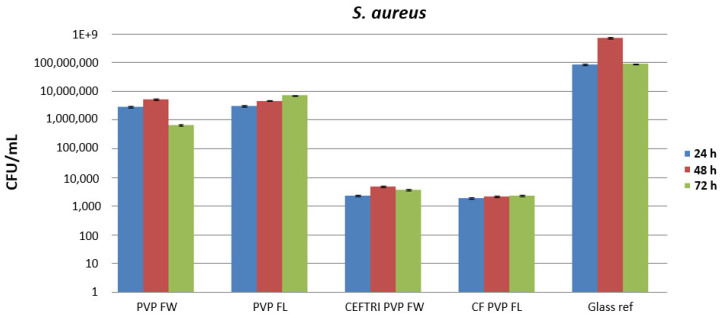
Biofilm formation results of the *S. aureus* tested strain, developed on nanomodified surfaces MAPLE-deposited at 200 mJ/cm^2^ laser fluence for 24, 48, and 72 h. Results represented on the Y-axis refer to log 10 CFU/mL.

**Figure 8:**
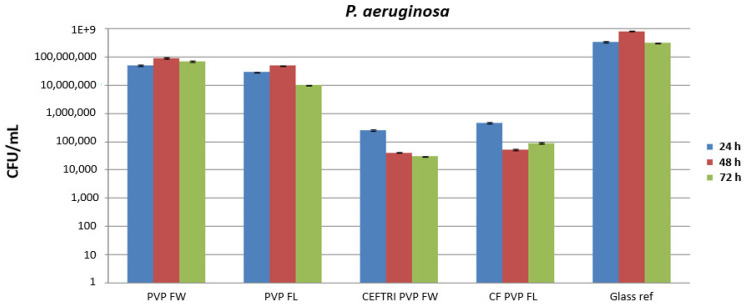
Biofilm formation results of the *P. aeruginosa* tested strain, developed on nanomodified surfaces MAPLE-deposited at 200 mJ/cm^2^ laser fluence for 24, 48, and 72 h. Results represented on the Y-axis refer to log 10 CFU/mL.

The antimicrobial and antibiofilm efficiency of the aforementioned experimental variants was evaluated using Gram-positive (*S. aureus*) (Figure 7) and Gram-negative (*P. aeruginosa*) (Figure 8) strains by the viable cell count assay and quantified at 24, 48, and 72 h.

In comparison to the glass reference that served as a control, the PVP/FW and PVP/FL composite thin films have a low inhibitory effect against *S. aureus* biofilm formation, both in early and late phases of microbial biofilm development (Figure 7). The corresponding CFU/mL values are quite similar in both the test samples and glass controls after 1 and 3 days of incubation (the fold change inhibition is up to 1 log in all cases) [50]. However, in case of CEFTRI/PVP/FW and CF/PVP/FL coatings, the number of biofilm-embedded viable cells significantly decreased after all time points of treatment, and the experimental data showed a decrease in CFU/mL values by more than three orders of magnitude. Even though a minor increase in biofilm formation is shown after 48 h in the case of CEFTRI/PVP/FW, all of the antibiotic/PVP/isoflavonoid thin films displayed superior anti-biofilm activity against *S. aureus*. These results confirm that flavonoids with poor pharmacokinetics and pharmacodynamics against microbes (when applied alone) can enhance the effect of antibiotics [63,64,65,66]. An interesting fact is that CF/PVP/FL thin films exhibited the same anti-biofilm efficiency against *S. aureus* over all of the incubation periods. This result indicates that the thin film can be active for at least three days.

At the 24 h incubation time, the reduction in the *P. aeruginosa* biofilm in the presence of all of the flavonoid-PVP thin films was lowered by a little over one order of magnitude, as compared with the glass reference. The most efficient combinations were the CEFTRI/PVP/FW and CF/PVP/FL coatings at the 24 h incubation time. CEFTRI/PVP/FW and CF/PVP/FL thin films exhibited similar inhibitory activity after 2 days of incubation, and both were associated with a decrease in the Gram-negative biofilm by almost 4 orders of magnitude. The most intense activity against *P. aeruginosa* biofilms was shown by CEFTRI/PVP/FW thin films, followed by CF/PVP/FL ones, and this effect was highly visible after 72 h of incubation time.

As observed from the microbiological data, the antibiotic/PVP/isoflavonoid thin films considerably reduced microbial colonization and biofilm formation of both *S. aureus* and *P. aeruginosa*, with more significant activity against *S. aureus*. These results suggest that isoflavonoids are favorable candidates for potentiating the activity of antibiotics. The PVP/isoflavonoid effects on Gram-positive and Gram-negative bacterial inhibition suggest a time- and strain-dependent tendency for biofilm formation. In both cases, the antimicrobial behavior could be attributed to the gradual release of isoflavonoids from PVP structures.

### 2.4. Biocompatibility Evaluation

**Figure 9:**
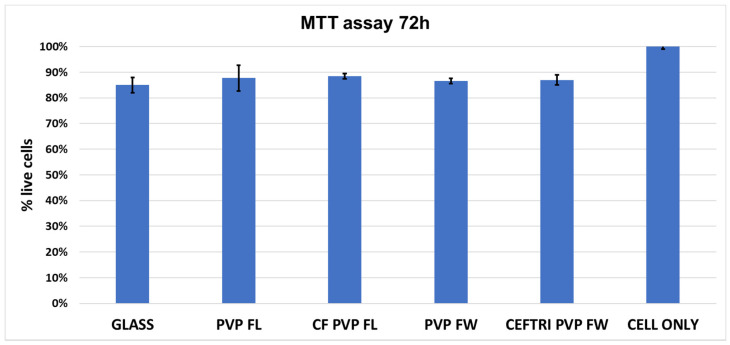
Cell viability of human endothelial cells after 72 h on the biopolymeric coatings.

The cytotoxic effect of isoflavonoid-antibiotic thin films was evaluated by measuring the metabolic activity of endothelial cells using the MTT assay. The biopolymeric coatings did not have a cytotoxic effect. The MTT assay demonstrated that after 72 h of incubation, human endothelial cells showed normal growth in the presence of all of the thin films, comparable to that of the control cells (Figure 9). The results are similar to those of other research groups that showed that all tested isoflavones induced the proliferation of endometrial stromal cells in a time- and concentration-dependent manner, starting at 48 h [67].

Smolińska et al. showed that isoflavone has anti-inflammatory properties in the human epithelial cell line HaCaT by inhibiting the cytokines IL-8, IL-20, and CCL2. In addition, they demonstrated that genistein prevented cytokine activation as well as TNF-α-induced NF-κB nuclear translocation, with no effect on the PI3K signaling, leading to the blockage of the NF-κB inflammatory signaling pathway [68].

**Figure 10:**
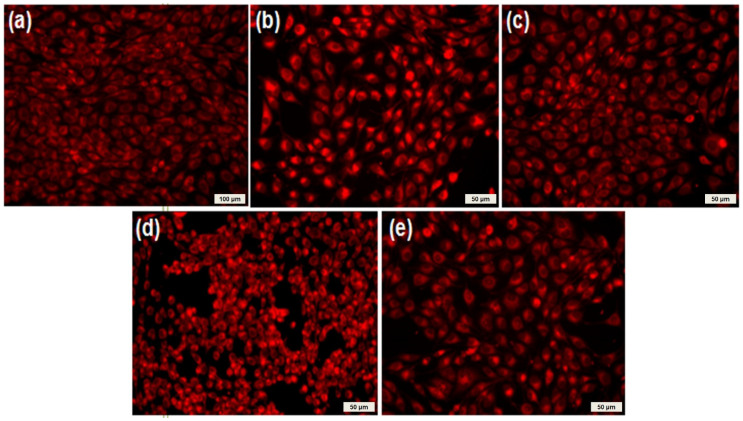
Microscopy images of human endothelial cells developed at 72 h on the surface of (**a**) glass control and (**b**) CF/PVP/FL, (**c**) PVP/FL, (**d**) CEFTRI/PVP/FW, and (**e**) PVP/FW MAPLE-deposited thin films. Viable endothelial cells were stained in red by Vital Cell tracker RED CMTPX fluorophore, showing that they are viable, with a normal morphology and adherence pattern.

After 3 days in the presence of isoflavonoid-antibiotic thin films, the endothelial cells showed normal morphology with endothelial-like characteristic appearance (Figure 10). Fluorescence images showed that the endothelial cells were viable—no dead cells or cell fragments were observed. Moreover, the cells formed filopodia to move and establish contacts with neighboring cells, suggesting that endothelial cells exhibited an active phenotype. A slight modification in the cell morphology was observed in the presence of the CEFTRI/PVP/FW thin films. The endothelial cells became round and withdrew their extensions, suggesting that the endothelial cells may have been affected (Figure 10d). This result may be due to the release of the combination of the two compounds.

## 3. Materials and Methods

### 3.1. Materials

PVP biopolymer, CF, and CEFTRI antibiotics as well as dimethyl sulfoxide (DMSO) solvent were purchased from Sigma-Aldrich. All chemicals were of analytical purity and used as received. Commercial, well-characterized FL and FW isoflavonoid powders (standards) were provided by Plantech UK.

### 3.2. Preparation of Mixes for Drop-Cast and MAPLE Depositions

Four different sterile solutions were used to coat the glass surface as follows:

i. Solution A: Cefuroxime:PVP (2% in DMSO):Luteone, 4:1:1 wt.% (CF/PVP/FL symbol).
ii. Solution B: PVP (2% in DMSO):Luteone, 1:1 w.t% (PVP/FL symbol).
iii. Solution C: Ceftriaxone:PVP (2% in DMSO):Wighteone, 4:1:1 wt.% (CEFTRI/PVP/FW symbol).
iv. Solution D: PVP (2% in DMSO):Wighteone, 1:1 wt.% (PVP/FW symbol).

### 3.3. MAPLE Experimental Conditions

All of the coatings were prepared using a COMPexPro 205 KrF* (λ = 248 nm and τ_FWHM_ = 25 ns) laser source from Lambda Physics-Coherent (Göttingen, Germany) that was operated at a fluence of 100–300 mJ/cm^2^, wherein 200 mJ/cm^2^ is the optimum laser fluence value. During deposition, all of the targets were maintained at a temperature of ∼173 K by active liquid nitrogen cooling and rotated to avoid heating and damage by the laser beam. The pulsed laser beam scanned the entire target surfaces at an angle of 45° with a repetition rate of 10 Hz for 100,000 subsequent laser pulses. All depositions were conducted at a substrate-to-target distance of 5 cm under a background pressure of 0.5 Pa. Thin coatings were deposited onto one-side polished Si (1 0 0) substrates and onto optical glass depending on the characterization methods to be used. Several solvents and laser parameters were revised in order to identify the optimal experimental conditions that allowed for the best compromise between the thin-film bioactivity and its morphology. For comparison purposes, drop-cast samples were prepared on Si (1 0 0) and glass substrates to evaluate the chemical structure of the thin films.

### 3.4. Characterization Methods

#### 3.4.1. Fourier-Transform Infrared Spectroscopy (FT-IR) and Infrared Microscopy (IRM)

FT-IR spectra and IRM mappings were recorded by means of a Nicolet iN10 MX FT-IR Microscope equipped with a MCT liquid nitrogen cooled detector (Thermo Fischer Scientific, Waltham, MA, USA). Spectral collection was made in reflection mode at 4 cm^−1^ resolution and over a measurement range of 4000–700 cm^−1^. For each spectrum, 32 scans were co-added and transformed to absorbance data using coupled OmicPicta software. For each sample, ~250 spectra were studied. In order to appreciate the chemical distribution of the main constituents of the thin films in the micro-domain ranges, IR maps were recorded.

#### 3.4.2. Scanning Electron Microscopy (SEM)

The morphological characterization of the thin films was undertaken using SEM, using a FEI Inspect S scanning electron microscope (Thermo Fisher Scientific, Hillsboro, OR, USA) at an acceleration voltage of 20 kV. Prior to SEM imaging, a thin gold layer was deposited onto sample surface to prevent the accumulation of electric charge.

#### 3.4.3. Antimicrobial Evaluation

For establishing the antimicrobial effect of the thin films, a monospecific biofilm model was used. For sample sterilization, we used a 15 W UV lamp incorporated into a safety laminar flow chamber (Asal Vertical Laminar Flow Hood Asalair Model 700, Milan, Italy). Briefly, samples of the thin films were sterilized under UV exposure (20 min on each side) and then placed in triplicate in 6 sterile well plates. Two mL of sterile nutritive broth was added to each material, and the prepared wells were seeded with 20 μL of freshly prepared microbial suspensions of 0.5 McFarland density (1.5 × 10^8^ CFU/mL) prepared from *S. aureus* ATCC 23235 and *P. aeruginosa* ATCC 27853 strains. The inoculated plates were incubated for various time intervals (24, 48, and 72 h) at 37 °C. After each incubation time, the samples were washed with sterile buffered saline to remove unattached cells, and the cells included in the biofilms were detached by vigorous stirring of the washed materials. Ten-fold dilutions were prepared from each sample containing biofilm-detached cells, and each dilution was inoculated in triplicate on nutritive agar Petri dishes. The plates were incubated for 24 h at 37 °C, and viable colony counts were performed. Each experiment was performed in triplicate and repeated on three separate occasions [69,70].

#### 3.4.4. Biocompatibility Evaluation

Endothelial cells (EAhy926 cell line, American Type Culture Collection, Manassas, VA, USA) were used to assess the biocompatibility and cytotoxicity of the isoflavonoid-antibiotic thin films. The cells were cultured in Dulbecco’s Modified Eagle Medium (DMEM), and they were supplemented with 10% fetal bovine serum and 1% penicillin (Gibco, Thermo Fisher Scientific, Waltham, MA, USA). In order to maintain the optimal culture conditions, the medium was changed twice a week. The cytotoxicity was assessed using the methyl-thiazol-tetrazolium (MTT) assay (Vybrant™ MTT Cell Viability Assay, Thermo Fisher Scientific, Waltham, MA, USA). The assay is a colorimetric method that allows for the quantitative evaluation of cell viability. The viable cells convert the water-soluble yellow tetrazolium salt MTT (3-(4,5-dimetiltiazoliu)-2,5-diphenyltetrazolium bromide)-insoluble purple formazan via mitochondrial enzymes. Briefly, the endothelial cells were grown in 24-well plates with a seeding density of 50,000 cells/well in the presence of isoflavonoid-antibiotic thin films for 72 h. Fifteen mL of Solution I was added and incubated at 37 °C for 4 h. Solution II was then added and vigorously pipetted to solubilize formazan crystals. After 1 h, the absorbance was read using a spectrophotometer at 570 nm (TECAN Infinite M200, Männedorf, Switzerland) [71].

The biocompatibility of endothelial cells was assessed by fluorescent microscopy using a RED CMTPX fluorophore (Thermo Fisher Scientific, Waltham, MA, USA), a cell tracker for long-term tracing of living cells. The dye was added after 5 days from endothelial cell cultivation in the presence of the isoflavonoid-antibiotic thin films. In order to allow for dye penetration into the cells, the RED CMTPX dye was added to the culture medium at a final concentration of 3 μM and then incubated for 30 min. The endothelial cells were washed with PBS three times and visualized by fluorescent microscopy. The photomicrographs were taken with a digital camera driven by Axio-Vision 4.6 software (Carl Zeiss, Oberkochen, Germany) [72].

#### 3.4.5. Statistical Analysis

The results were statistically analyzed using GraphPad Prism Version 5.04 for Windows (GraphPad Software, San Diego, CA, USA). For comparison, we used the number of colony-forming units per mL (CFU/mL), as revealed by the readings of three values/experimental variants. Logarithmic values were used for statistical analysis. We chose to employ the two-way ANOVA and Bonferroni test for revealing significant differences among the analyzed groups (*p*-values lower than 0.05 were considered to be significant).

## 4. Conclusions

We have successfully deposited highly biocompatible isoflavonoid-based composite thin films by MAPLE with excellent anti-adherence and antibiofilm effects against both Gram-negative and Gram-positive bacterial strains, allowing for both normal development and growth of human endothelial cells. FT-IR and IRM showed the chemical bonding and the homogeneity of the antibiotic/PVP/isoflavonoid thin films, while SEM micrographs revealed the uniform morphologies of the MAPLE thin films. Microbiological data showed that the MAPLE thin films efficiently inhibited *S. aureus* and *P. aeruginosa* adherence and biofilm formation for all of the tested time points. These results demonstrated that the antibiotic/PVP/isoflavonoid thin films deposited by MAPLE can be successfully applied to improve the resistance to microbial colonization and prevent the development of healthcare-associated infectious diseases. This work highlights the therapeutic potential of natural isoflavonoids to enhance the antimicrobial activity of conventional antibiotics. Therefore, these results have emphasized the potential application of bioactive composite systems and the MAPLE deposition approach for the development of innovative, safe, and green antimicrobial strategies for improved medical devices and contact surfaces.

## Data Availability

The data presented in this study are available on request from the corresponding author.

## Sample Availability

Samples of the compounds are available from the authors.
